# First-Principles Dynamics of the Surface Fluorination of Diamond

**DOI:** 10.1021/acs.langmuir.5c01587

**Published:** 2025-06-18

**Authors:** Henry Thake, Stephen J. Jenkins

**Affiliations:** Yusuf Hamied Department of Chemistry, 117213 University of Cambridge, Lensfield Road, Cambridge CB2 1EW, U.K.

## Abstract

We present first-principles dynamic simulations of molecular fluorine dissociatively adsorbing on clean C{001} and identify a range of possible outcomes depending upon the interplay of impact site, molecular orientation, and surface temperature. These include adsorption of a single fluorine atom with desorption of the other, in which case both the surface and the isolated atom gain radical character. In most scenarios, however, both fluorine atoms adsorb, either at opposite ends of a single surface dimer or on two such dimers. In the former situation the reaction end-point is closed-shell in nature, whereas in the latter the surface acquires singlet diradical character.

## Graphical abstract

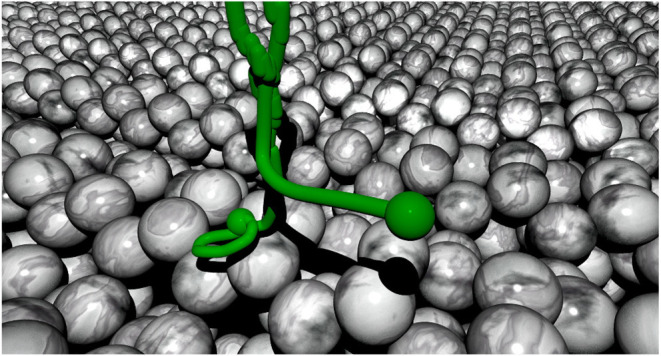

## Introduction

Alongside its exceptional stability and near-unrivalled thermal conductivity, the wide band gap of diamond makes it an extremely attractive proposition for high-temperature semiconductor applications. Set against these favorable properties, however, the difficulty of controllably doping bulk diamond has hampered efforts to fabricate practical devices, leading to significant interest in the concept of surface transfer doping.^1^ In this scenario, adsorbed species modify the surface dipole and hence the substrate’s electron affinity. This, in turn, injects free carriers into the conduction or valence band, with useful concentrations being achievable in sufficiently thin diamond films.

One key parameter determining the efficacy of surface transfer doping is the polarity of the adsorbate–substrate bond, implying that strongly electropositive or electronegative species are likely to be good options. For this reason, fluorine overlayers on diamond have been studied in this connection, by both experimental^2–7^ and theoretical^8–14^ means. We note in passing that fluorinated diamond surfaces are also believed to display remarkable low-friction and hydrophobic qualities,^2^ making them ideal for many potential sensor applications. Most often, deposition of fluorine on diamond is achieved via exposure to highly reactive fluorine atoms or to fluorine-containing radicals and/or plasma, but on occasion exposure to molecular fluorine gas has been considered instead – see, for example, a recent study on hydrogen-terminated polycrystalline diamond.^15^ This would have the advantage of reducing the likelihood of etching, contaminating, or otherwise damaging the diamond surface. Set against this, the unreactive nature of diamond implies that the action of molecular fluorine will be comparatively slow and inefficient, especially if the surface is not initially clean but somewhat hydrogenated.^2^

As a point of reference, we might mention here the superficially similar case of silicon fluorination. One of us recently reported first-principles dynamic simulations of molecular fluorine adsorption on clean and hydrogen-passivated Si{001} in which all bar one of the computed trajectories resulted in fluorine deposition.^16^ Even the single exception, which occurred on the monohydrogenated surface, generated two clean surface sites (via HF desorption) that would be ripe for subsequent adsorption. The key difference in the case of diamond, however, lies in the nature of its surface dimers and dangling bonds.

It is well-known that the {001} surfaces of carbon, silicon, germanium and tin exhibit rather similar reconstructions, based upon the formation of surface dimers.^17^ In all but the first of these materials, formation of the σ-like dimer bond simply saturates one dangling bond from each of two neighboring top-layer atoms, leaving a second dangling bond on each atom in a semioccupied condition. The existence of semioccupied dangling bonds being inherently unstable, however, electron transfer across the dimer results in its buckling into an asymmetric conformation (essentially a Jahn–Teller distortion) with the dangling bond on the higher-lying atom becoming filled and that on the lower-lying atom becoming empty. These dangling bonds nevertheless remain highly susceptible to reaction with incoming species, with which they readily form covalent bonds.

In diamond, however, the σ-bonds are at least 34% shorter than in the other elemental solids of the carbon group (ultimately a consequence of carbon being a 2p element). Accordingly, those dangling bonds not involved in the dimer’s σ-bond substantially overlap and contribute a π component to what is consequently a symmetric dimer. That is, the dimer bond in diamond effectively displays double-bond character, which is indeed reflected in its much shorter length compared with single bonds in the bulk material (1.34 Å versus 1.57 Å in our calculations). The consequent absence of genuine dangling bonds on the C{001} surface leads us to expect a lower propensity for the dissociative adsorption of molecular fluorine than was found for Si{001} but just how much lower cannot be predicted without conducting comparative simulations. That is the purpose of the present work.

In contrast to previous computational studies that have predominantly reported on structural and electronic properties of static fluorine monolayers,^12,13^ or quasistatic transition-state treatments of adsorption,^14^ we here report on first-principles molecular dynamic simulations. Such an approach has seen increasing use in studies of surface scattering, adsorption, diffusion, reaction and desorption,^18–41^ permitting one to follow detailed trajectories for individual events, albeit at significant computational cost. We are necessarily limited, therefore, to calculating a relatively small number of representative trajectories, but can nevertheless expect to gain mechanistic insight lacking from alternative methods. Furthermore, we are able directly to address the role of surface temperature in determining the reactive channels accessed by incoming molecules.

## Computational Method

First-principles density functional calculations were performed, subject to periodic boundary conditions, using the CASTEP computer code (Version 18.1).^42^ Parameters were very similar to our group’s previous investigation into the dynamics of fluorine adsorption on silicon,^16^ to which the interested reader is directed for comparative details. In summary, however, we once again used a simulation cell consistent with a c(4 × 4) unit cell in the surface-parallel plane, of length in this instance equivalent to 24 layers of diamond in the [001] crystallographic direction. Within this supercell, the surface was represented by a slab comprising eight carbon layers, of which the uppermost was reconstructed into a (2 × 1) array of symmetric dimers. The outermost layer of the back surface, meanwhile, was not only dimerized but also passivated with a single hydrogen atom attached to each carbon atom. All but the sixth and seventh carbon layers were then permitted to relax according to the calculated forces. Convergence in our initial geometry optimization calculations was accepted when the energy change per iteration fell below 10^–8^ eV/atom; the corresponding maximum atomic displacement fell below 10^–2^ Å; and the forces all fell below 2 × 10^–2^ eV·Å^–1^. Electronic wave functions were represented throughout within a basis set of plane waves, up to a kinetic energy cutoff at 350 eV, while sampling of the Brillouin zone was achieved through a 3 × 3 × 1 Monkhorst–Pack mesh.^43^ Electron–ion interactions were included through use of ultrasoft pseudopotentials,^44^ and the exchange-correlation interactions between electrons were included through the Perdew–Burke–Ernzerhof functional.^45^ The system was treated as potentially metallic, and spin was unconstrained. Semiempirical dispersion corrections were included through the Tkatchenko-Scheffler scheme.^46^

For the dynamic simulations, the back three carbon layers and passivating hydrogen atoms were frozen in place and a time-step of 0.5 fs was adopted within the NVE ensemble (i.e., atom numbers, supercell volume, and total energy were all held constant). Electronic convergence at each step was judged against tolerances of 10^–5^ eV per atom for the total energy and 10^–6^ eV for individual eigenvalues. The incoming molecule was initialized with its center of mass at a height of 6.0 Å above the top-layer carbon atoms of the relaxed surface, traveling downward along the surface normal at a speed of 362 m·s^–1^ (kinetic energy 0.026 eV) corresponding to the most probable speed for gaseous F_2_ at 300 K (N.B. two thirds of the speed corresponding to the most probable molecular kinetic energy at this temperature).

Pre-empting our discussion of results only slightly, we mention here that transfer of electronic charge from the surface to the molecule induces vibrations in the latter from the very start of each trajectory. This is because each individual simulation begins as if the molecule were suddenly transported from a distant location to its starting point in an instantaneous (adiabatic) manner. To avoid these vibrations, one might imagine prerelaxing the molecule while fixing its center-of-mass at some position above the surface, but this scenario is equally unphysical, because it implies that the molecule is transported to its starting location quasistatically (isothermally). Reality lies somewhere in-between, and the only true solution to this dilemma is to start each simulation with the molecule sufficiently distant from the surface that both scenarios coincide. This is not, however, a practical proposition and one must commit to one or the other approach – for us, this is the adiabatic one. In all other respects, our simulations most closely resemble supersonic molecular beam experiments, in which molecules approach the surface in a tightly collimated manner, with a very narrow spread of translational energy, and in a rotationally cold state. This justifies us in commencing all of our trajectories with purely vertical, monoenergetic, and rotation-free molecular motion.

In each such trajectory, the molecule was aimed at one of five nominal target sites (A–E) that span the surface unit cell in uniform fashion (see Figure 1). For each target site, simulations were initialized with the molecule aligned having its axis parallel to one of 13 possible directions, defined by combinations of three unit vectors forming a right-handed orthogonal set and lying: (α) along the dimer row direction; (β) across the dimer row direction; and (γ) along the outward surface normal. The notation αγ, for example, implies the direction resulting from summation of the α and γ vectors, while αβγ indicates the summation of the α vector with the difference between the γ and β vectors (the overline indicating negation). In this way, we obtain a total of 65 trajectories, but consideration of symmetry reduces this to just 41 distinct instances that actually require computation (see Table [Table tblI]). In order to model the surface at two different temperatures, the substrate atoms were initialized either (a) in their relaxed geometry at 0 K, or (b) after equilibration of the clean surface for 1 ps in a separate NVT calculation with temperature fixed at 300 K. We note that dissociative adsorption of F_2_ is likely to liberate sufficient energy to dwarf any thermal energy thus included in the upper layers of our system, but nevertheless anticipate that it may be possible for stochastic motion to send individual trajectories into different reaction channels in the higher-temperature scenario.

**Figure 1:**
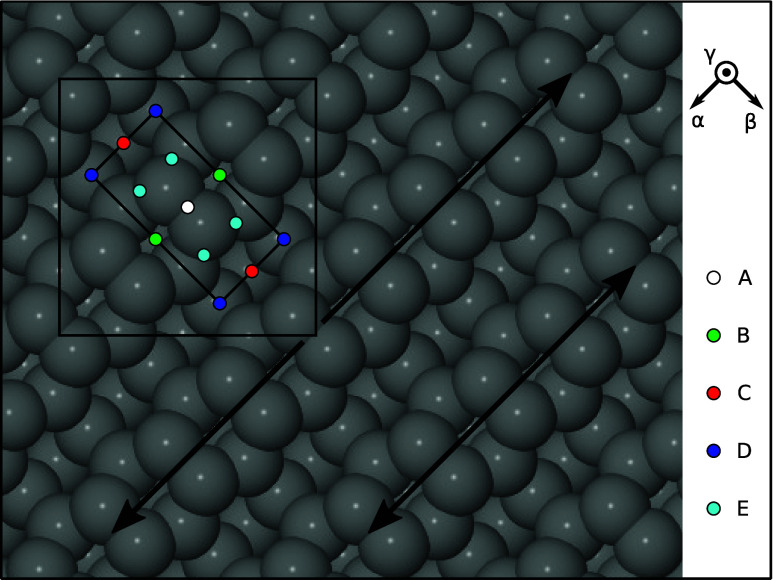
Schematic top-down view of the C{001} surface, highlighting two adjacent dimer rows with double-headed arrows. The surface exhibits a (2 × 1) reconstruction, whose primitive cell is marked with the inner rectangle, but calculations were performed within a c(4 × 4) unit cell, marked with the large square. Incoming molecules were aimed at sites A–E, shown here in multiple instances to emphasize the uniformity of their distribution; for complete specification, the left-most of the indicated E sites was used for the calculations. Reference axis orientations are indicated by vectors labeled α (along the dimer rows) and β (across the dimer rows). A third orientation, γ, corresponds to the outward surface normal.

**1 tblI:** Initial Molecular Orientations, Labelled According to the Direction of the Molecular Axis with Respect to Surface-Related Axes (α, β, and γ Defined as in Figure 1)­[Table-fn tIfn1]

site	primary	symmetry-related
A–D	α	
	β	
	γ	
	αβ	αβ
	αγ	αγ
	βγ	βγ
	αβγ	αβγ, αβγ, αβγ
E	α	
	β	
	γ	
	αβ	
	αβ	
	αγ	
	αγ	
	βγ	
	βγ	
	αβγ	
	αβγ	
	αβγ	
	αβγ	

a Under the symmetry-related heading, we note orientations that are equivalent to those listed in the primary column through some combination of mirror or rotational symmetry operations. Entries listed under the primary column comprise 41 symmetrically distinct site/orientation combinations; including the symmetry-related column raises this to 65 in total.

For interpretative purposes, we mainly rely upon plots of interatomic separation, noting especially those instances when distances deviate significantly from the equilibrium bond lengths summarized in Table [Table tblII]. When separations oscillate around these bond lengths, we estimate vibrational stretch frequencies directly from the periodic spacing of turning points in the separation plot, quoting results only to the nearest multiple of 5 cm^–1^ after averaging across at least five successive maxima or minima. Where a sufficiently regular sequence of turning points is not readily identifiable in the data, we decline to estimate a frequency for the corresponding mode. Identification of bond formation and cleavage is further aided, however, by keeping track of two different spin measures as each trajectory progresses.

**2 tblII:** Equilibrium Bond Lengths Used as a Benchmark for Identifying Bond Making and Breaking[Table-fn tIIfn1]

bond	notes	length (Å)
F–F	(calculated gas phase)	1.42
F–C	(F attached to C dimer)	1.35
C–C	(typical single bond)	1.54
C=C	(typical double bond)	1.34

a The first two values are computed in this work; the latter two are based on the experimental literature.

First, we plot the integrated net spin, defined as

1 $$\sigma_{1}=\left|\int \left(\rho_{\alpha }\left(r\right)-\rho_{\beta }\left(r\right)\right)\mathrm{dr}\right|$$

where ρ_α_(**r**) and ρ_β_(**r**) are the spin densities of the two spin species accounted for in our calculations, and where the integral spans the full volume of the supercell. Note that the spin labels α and β should not be confused with the axis labels used in defining molecular orientations above. Since our calculations do not include spin–orbit interactions, the spin directions are strictly nominal and do not correspond to any particular crystallographic axes.

Second, we also plot the integrated spin modulus, defined as

2 $$\sigma_{2}=\int \left|\rho_{\alpha }\left(r\right)-\rho_{\beta }\left(r\right)\right|\mathrm{dr}$$

in which the modulus of the integrand is taken, rather than that of the integral. The integrated net spin, σ_1_, will only be nonzero when the system possesses a well-defined majority spin species, whereas the integrated spin modulus, σ_2_, will be nonzero whenever the system possesses regions of local spin imbalance, even if multiple such regions counterbalance one another perfectly when considered together. In the cases described below, we shall often see instances where σ_2_ approaches a value of 2 μ_B_, indicating that the system possesses effectively two free spins. If σ_1_ achieves a similar value at the same time, we may infer that these spins align parallel with one another, effectively forming a triplet state, while if σ_1_ remains close to zero then a singlet state, in which these spins are antiparallel, must be inferred. For a thorough discussion of such diradical systems, the interested reader is referred to the recent insightful review article by Stuyver et al.^47^

## Results and Discussion

Surveying the simulations performed with an initial surface temperature of 0 K, all but one resulted in some degree of adsorption. The sole exception was the B/γ trajectory, in which the molecule bounced off the surface with no C–F separation falling below 2.34 Å at any stage. A second trajectory, labeled D/γ, almost follows the same pattern, with the molecule bouncing off the surface but being captured by dispersion forces and returning for a second bounce before eventually reacting on its third attempt. In fact, however, these trajectories retain a high degree of symmetry throughout their initial approach phase, suggesting that nonreactive scattering is unlikely to occur frequently outside of our somewhat idealized calculations. We shall therefore bestow no further attention upon these cases and proceed instead to classify the reactive trajectories according to their eventual end-points following dissociative adsorption.

Multiple examples may be found among our trajectories for the following types of adsorption: (I) a single fluorine atom bonds to a single top-layer carbon atom, while the other fluorine atom is ejected from the surface region; (II) both fluorine atoms attach separately to either end of a single carbon dimer; (III) the fluorine atoms bond separately with carbon atoms belonging to a pair of dimers that are neighbors within the same dimer row; (IV) the fluorine atoms bond separately with carbon atoms belonging to dimers that are neighbors with respect to adjacent dimer rows; and (V) the fluorine atoms bond to dimers that are not neighbors at all, either within the same row or between two adjacent rows. We further subdivide the third and fourth categories according to whether the adsorbed fluorine atoms on neighboring dimers lie proximal to one another or not, yielding categories that we label IIIa and IVa (where the fluorine atoms are proximal to one another) or IIIb and IVb (where they are not). The results of all trajectories at this surface temperature are summarized in Table [Table tblIII] and a histogram is provided in Figure 2. Outcomes are distributed fairly evenly between Types I–IV, albeit with a notable paucity of Types II and IVb. The apparent preference for an outcome of Type V should be interpreted with caution, given that we have not subdivided this category.

**3 tblIII:** Classification of the Trajectories Calculated in the Present Work at an Initial Surface Temperature of 0 K[Table-fn tIIIfn1]

*T* = 0 K	A	B	C	D	E
α	IIIa	IIIb	V	V	IIIa
β	II	IIIb	IVa	V	IVa
γ	I	N	IVa	IVb	I
αβ	II	IIIb	IVa	V	IIIa
αβ	II	IIIb	IVa	V	V
αγ	I	IIIa	V	V	V
αγ	I	IIIa	V	V	IIIa
βγ	I	IIIb	IVa	V	V
βγ	I	IIIb	IVa	V	IIIa
αβγ	I	IIIb	V	V	V
αβγ	I	IIIb	V	V	IIIa
αβγ	I	IIIb	V	V	I
αβγ	I	IIIb	V	V	IIIa

a Column headings refer to the adsorption sites at which the molecule is initially aimed (cf. Figure 1) while row headings define its orientation (cf. Table [Table tblI]). Roman numerals then indicate the type of reaction observed, as per the typology described in the main text. The trajectory marked “N” led to no adsorption.

**4 tblIV:** Classification of the Trajectories Calculated in the Present Work at an Initial Surface Temperature of 300 K[Table-fn tIVfn1]

*T* = 300 K	A	B	C	D	E
α	IIIa	IIIa	V	IIIa	IIIa
β	II	II	IVa	IVa	IVa
γ	I	IIIb	IVa	I	V
αβ	II	IIIb	IVa	V	V
αβ	II	IIIb	IVa	V	V
αγ	I	IIIa	I	IIIa	I
αγ	I	IIIa	I	IIIa	V
βγ	II	IIIb	I	V	V
βγ	II	IIIb	I	V	V
αβγ	II	IIIb	IVa	V	I
αβγ	II	IIIb	IVa	V	IIIa
αβγ	II	IIIb	IVa	V	IIIb
αβγ	II	IIIb	IVa	V	V

a Column headings refer to the adsorption sites at which the molecule is initially aimed (cf. Figure 1) while row headings define its orientation (cf. Table [Table tblI]). Roman numerals then indicate the type of reaction observed, as per the typology described in the main text.

**Figure 2:**
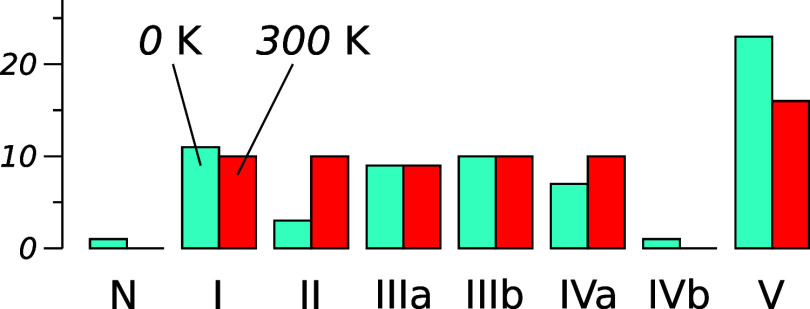
Histogram of reaction types observed in simulations conducted with initial surface temperatures of 0 and 300 K. Roman numerals correspond to the typology described in the text (see also Tables [Table tblIII] and [Table tblIV]).

Reviewing Table [Table tblIII] in more detail, it is noteworthy that reactions of Type I are highly correlated with trajectories aimed toward the A site, as are the very few reactions of Type II, while Types IIIa and IIIb are similarly correlated with trajectories targeting the B site, albeit showing some lesser association with the E site. Type IV outcomes are almost exclusively associated with trajectories in which the incoming molecule is aimed at the C site – one of only two exceptions being the atypical D/γ trajectory alluded to above (also the only example leading to a Type IVb end-point). Reactions of Type V are highly correlated with trajectories targeting site D and, to a somewhat lesser extent, sites C and E. The pattern of reactivity is clearly far from random at this surface temperature.

Turning to simulations performed with an initial surface temperature of 300 K, the picture remains broadly similar to, but not identical with, that revealed above for the cryogenic surface. First, we note that the reaction end-point, summarized in Table [Table tblIV], is unchanged in 35 out of our total complement of 65 trajectories (21 out of the 41 symmetrically distinct instances) from the equivalent entries in Table [Table tblIII]. We shall describe a selection of these cases in the subsections below, illustrating just how similar the cold and warm trajectories really are.

Second, the redistribution of end-point types among the other 30 trajectories (20 of them being symmetrically distinct) becomes somewhat more uniform than for the cryogenic surface, owing to a marked increase in the number of Type II cases and a notable reduction in Type V cases (see Figure 2). This is not, however, the result of a straight swap between these two categories. In fact, the additional Type II trajectories were almost all Type I trajectories in the 0 K simulations, and none were previously of Type V. The number of Type I trajectories falls only very slightly, however, because of conversion from Type IVa, Type IVb, and especially Type V pathways. Meanwhile, other trajectories of Type V convert either to Type IIIa or to Type IVa, so the overall pattern is clearly quite complex and rather contingent. Nevertheless, the trend is toward more closely spaced adsorption sites in the higher-temperature regime.

Third, despite the changes described above, there remains at 300 K a significant degree of structure in the distribution of reaction end-points among trajectories aimed at certain surface sites. Reviewing Table [Table tblIV] in detail, we see that reactions of Type II are highly correlated with trajectories aimed toward the A site, while those of Types IIIa and IIIb remain similarly correlated with trajectories targeting the B site. The association between reactions of Type IVa and trajectories in which the incoming molecule is aimed at the C site remains just as strong as when the surface started at 0 K. The distribution of Type I reactions has, however, lost its tight association with trajectories in which the molecule is aimed at site A, with site C now being more conducive to such an end-point, alongside sites A, E, and D in order of decreasing instances. Meanwhile, reactions of Type V are now almost exclusively confined to cases where the molecule was aimed at either site D or site E, with only a single example remaining where it was aimed at site C.

In short, the effect of surface temperature on the adsorption of fluorine is relatively subtle. We stress that while some atypical behavior seen at 0 K is absent from the 300 K results (specifically, nonreactive scattering) it is notable that no *new* phenomenology arises at the higher temperature. Thermal motion of the substrate merely deflects around half of the calculated trajectories into different channels plucked from among a set of archetypes recognizable from those in which the surface started off cold. Probabilities of entry into particular channels from particular starting configurations may vary, but the behavior within each channel is relatively insensitive to surface temperature. Quite how insensitive will now be addressed through examination of individual case studies, including examples of six possible reaction end-points. Specifically, we have chosen cases where the same starting configuration leads to the same end-point at both studied surface temperatures, in order better to illustrate similarities and differences. In each instance, plots of interatomic separation and spin characteristics will be discussed, in which thick lines relate to simulations based on an initial surface temperature of 300 K and thin lines relate to simulations based on an initial surface temperature of 0 K.

### Type I Trajectory

The first of these plots is presented (for the A/*αγ* case) in Figure 3, which shows the C–F separation between the initially lower-lying fluorine atom and the carbon atom to which it will eventually bond (upper panel, blue trace) together with the C–F separation between the initially higher-lying fluorine atom and the carbon atom to which it most closely approaches (upper panel, red trace). The two carbon atoms involved in these separations are, in fact, at opposite ends of a single surface dimer. As the simulation progresses, both separations show signs of acceleration toward the surface, while the F–F distance (middle panel, green trace) fluctuates, indicating onset of molecular vibration in response to a degree of electron transfer from the substrate. Both the integrated net spin (lower panel, magenta trace) and the integrated spin modulus (lower panel, black trace) initially fluctuate in sympathy, for the same reason, indicating nascent triplet character. After about 200 fs, however, the former quantity tends toward zero (in both simulation) while the latter periodically approaches 1 μ_B_ whenever the molecule nears its transition state for dissociation.

**Figure 3:**
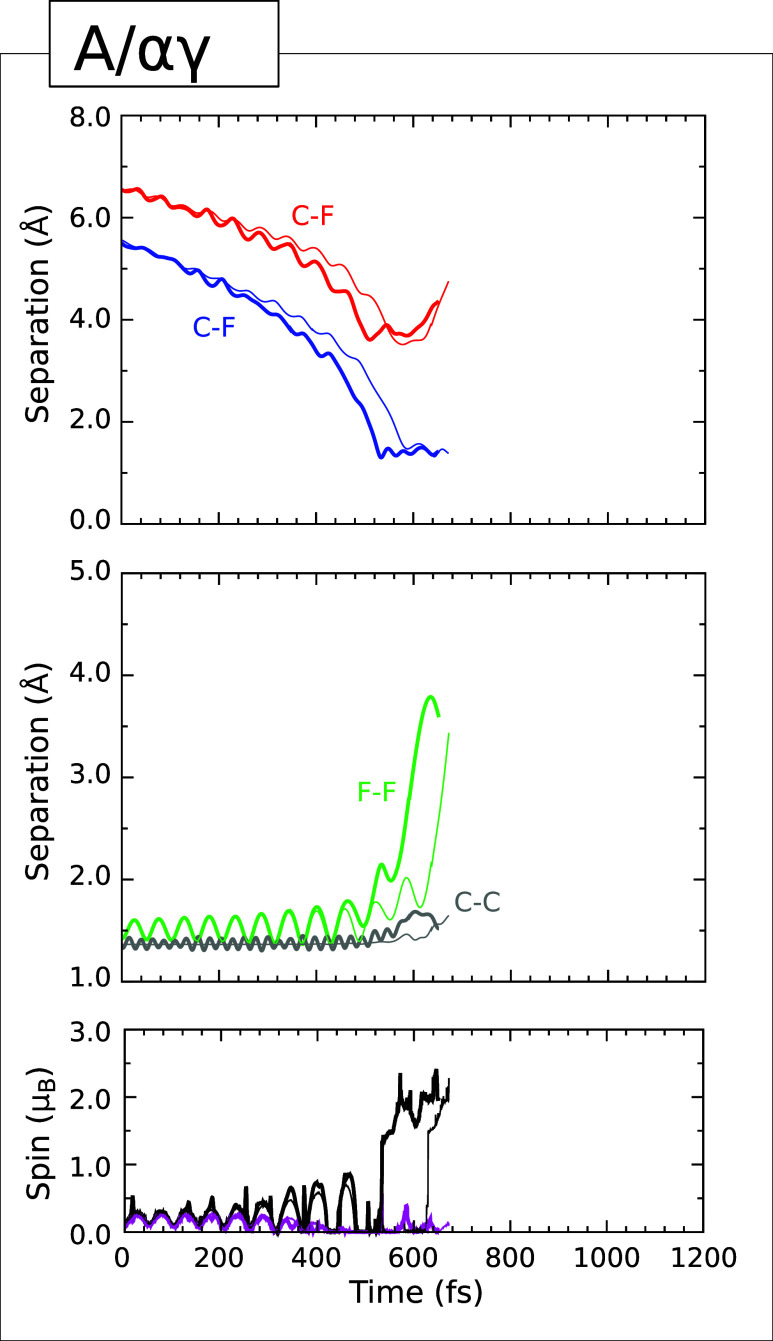
Evolution of the A/αγ (Type I) trajectory. The upper panel shows the C–F separation corresponding to the adsorption of one fluorine atom (blue) together with the shortest C–F separation for the desorbing fluorine atom (red). The middle panel shows the F–F separation (green) and the C–C separation for the dimer to which one of the fluorine atoms becomes attached (gray). The lower panel shows the integrated net spin (magenta) and the integrated spin modulus (black) Across all panels, simulations performed with an initial surface temperature of 0 K (300 K) are indicated with thin (thick) traces.

In the 300 K simulations, the critical moment occurs at around the 535 fs mark, when the C–F separation involving the lower-lying fluorine atom (upper panel, blue trace) reaches its minimum value of 1.30 Å. At this time, the F–F separation achieves a temporally local maximum of 2.15 Å (middle panel, green trace) and the integrated spin modulus (lower panel, black trace) jumps to a value around 1.5 μ_B_. Over the next 115 fs, the nascent C–F bond settles into oscillations within the range 1.29–1.51 Å, while the C–C length of the involved dimer fluctuates within the range 1.43–1.69 Å, consistent with a single bond (cf. the bond length of 1.36 Å about which it oscillated prior to reaction). The F–F separation, meanwhile, increases to in excess of 3.60 Å and the shortest C–F distance involving the higher-lying fluorine atom exceeds 4.35 Å by the end of the simulation at just beyond the 650 fs mark. Indeed, this fluorine atom ultimately achieves a height of 4.37 Å (relative to the initial mean vertical position of the top-layer carbon atoms) at a speed of 909 m·s^–1^, of which the surface-normal velocity component amounts to 874 m·s^–1^. That is to say, the kinetic energy of this single fluorine atom (0.082 eV) exceeds that initially supplied to the fluorine molecule (0.026 eV) by a factor of more than three, while the fraction attributable to the surface-normal translational mode amounts to 0.076 eV. Since this atom has already substantially departed from the surface, it seems highly debatable whether any dispersion forces remaining at this distance would be sufficient to bring it back. Unfortunately, our simulations of trajectories with Type I end-points all run into substantial convergence issues at around this juncture, so it is impractical to continue further.

Considering all of the Type I trajectories that started with a cryogenic surface, the median height of the departing fluorine atom at termination was 4.18 Å with a median vertical velocity component of 542 m·s^–1^, while for the Type I trajectories that started with a warm surface, the median height at termination was 3.94 Å with a median vertical velocity component of 780 m·s^–1^ (see Table [Table tblV]). Mean values of these parameters are quite similar to the median values for the 300 K simulations, but are considerably skewed upward by a few very energetic trajectories in the 0 K data set, including especially the A/αγ trajectory. By contrast, however, the end-point of the 300 K version of this trajectory, described in detail within the present subsection, is somewhat more energetic and distant from the surface than the median, but not dramatically so. Noting the variability among the Type I trajectories, we nevertheless believe that several are probably consistent with complete desorption of radical fluorine, while in other cases this species may eventually return to the surface after a protracted but ultimately transient solitary interlude.

**5 tblV:** Parameters Relating to the Departing Fluorine Atom at the End of All Symmetrically Distinct Type I Trajectories[Table-fn tVfn1]

0 K		*h* (Å)	|*v*| (m·s^–1^)	*v*_⊥_ (m·s^–1^)	*KE*_⊥_ (eV)
A/γ	(1)	4.66	551	542	0.029
A/αγ	(2)	4.67	2971	2971	0.876
A/βγ	(2)	4.18	1646	1607	0.256
A/αβγ	(4)	4.09	335	236	0.006
E/γ	(1)	4.08	1220	529	0.028
E/αβγ	(1)	4.76	1216	652	0.042
mean	(11)	4.32	1233	1075	0.217
median	(11)	4.18	1216	542	0.029

300 K		*h* (Å)	|*v*| (m·s^–1^)	*v*_⊥_ (m·s^–1^)	*KE*_⊥_ (eV)
A/γ	(1)	4.55	982	750	0.056
A/*α*γ	(2)	4.37	909	874	0.076
C/αγ	(2)	3.92	1878	941	0.088
C/βγ	(2)	3.51	2147	727	0.052
D/γ	(1)	3.95	995	738	0.054
E/αγ	(1)	3.91	1531	294	0.009
E/αβγ	(1)	4.00	2243	810	0.065
mean	(10)	4.00	1562	768	0.062
median	(10)	3.94	1705	780	0.061

a Height, *h*, is measured relative to the initial mean vertical position of the top-layer carbon atoms. Speed and the surface-normal component of velocity are denoted |*v*| and *v*_⊥_ respectively, while *KE*_⊥_ represents the kinetic energy associated with the latter velocity component. Mean and median values are computed by weighting the entries in each column by their multiplicities, indicted in parentheses.

Significantly, however, we note that all of our Type I trajectories terminate with an integrated spin modulus value of around 2 μ_B_ and an integrated net spin of zero. Examination of the 300 K system’s net spin density at the 650 fs mark, as displayed in Figure 4, reveals localization on the departing fluorine atom and on the unsaturated carbon atom from the dimer to which the other fluorine atom is attached. Mulliken analysis^48^ indicates spin moments of −0.67 and +0.86 μ_B_ respectively, qualitatively consistent with a semioccupied p orbital on the fluorine atom and a semioccupied sp^3^ dangling bond on the carbon atom. Both the desorbing species and the surface itself may therefore be regarded as possessing substantial radical character.

**Figure 4:**
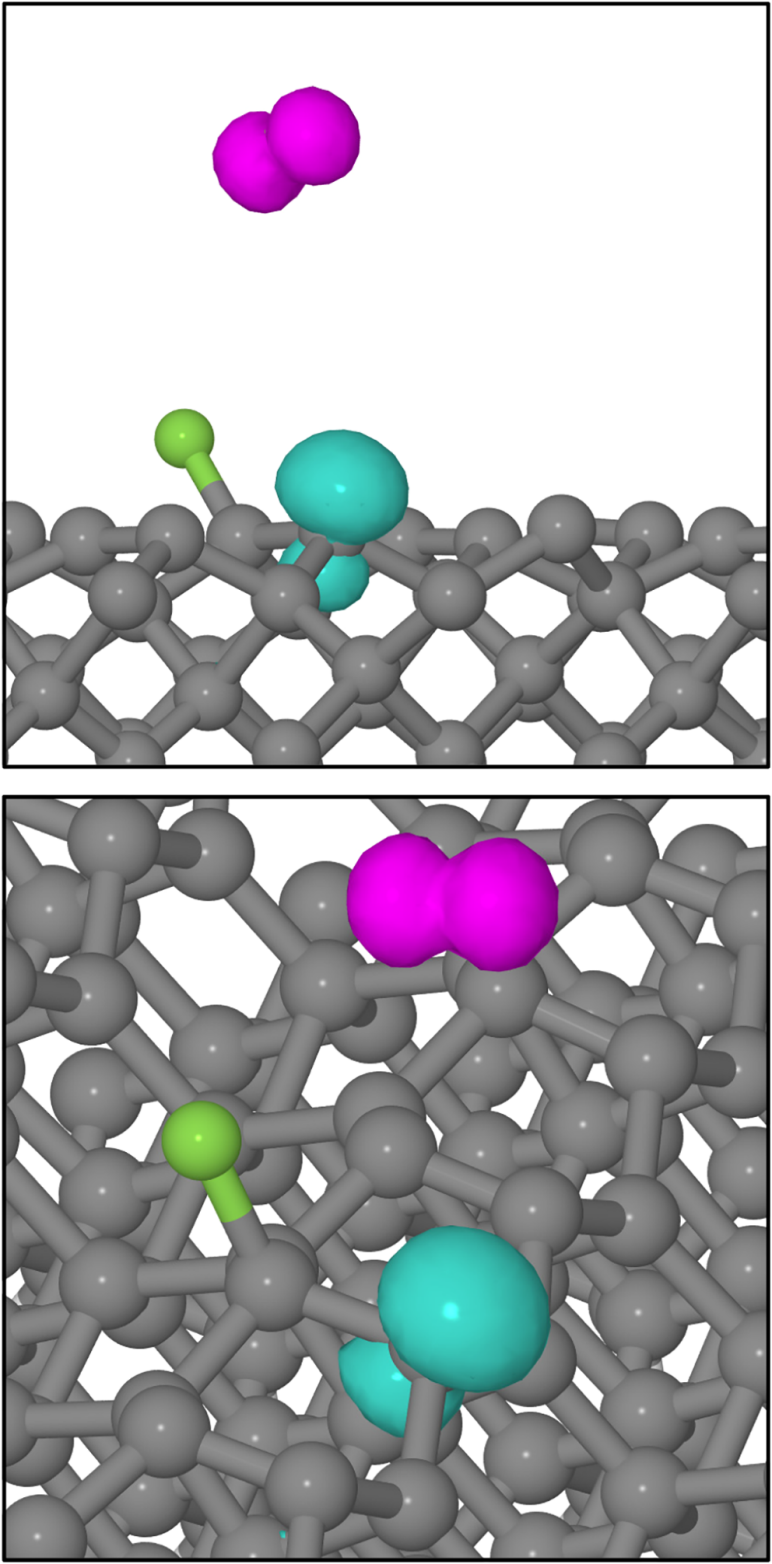
Net spin density at the end of the A/αγ trajectory (isosurfaces plotted at 0.1 μ_B_·Å^–3^ with turquoise and magenta indicating opposing signs). The upper panel shows an orthographic projection along the [110] crystallographic direction, while the lower panel shows a view from an elevated position along a similar azimuth. Gray and green spheres represent carbon and fluorine atoms, respectively. Note the p-like distribution of spin on the isolated fluorine atom, and the sp^3^-like distribution at the carbon dangling bond.

As may be seen from Figure 3, the evolution of the A/αγ trajectory progresses in very similar manner when the surface is initialized at 0 K, the only significant difference being that all of the key events discussed above are delayed by around 50–60 fs. The warm surface seems thus to be ever so slightly more reactive than the cold, at least in this particular instance.

### Type II Trajectory

The evolution of key interatomic separations and spin measures for the A/αβ trajectory is presented in Figure 5. Here, the symmetry of the flat-lying molecular geometry happens to dictate that the two shortest C–F distances (upper panel, blue and red traces) are initially identical, and so they more-or-less remain throughout both simulations. This is especially apparent when starting with a surface temperature of 0 K (thin lines) but is also substantially true when starting with a surface temperature of 300 K (thick lines). Focusing on the latter simulation, we observe progressive acceleration of the molecule toward the surface, culminating in a moment of simultaneous closest approach at the 690 fs mark, when both C–F separation achieve a value of 1.21 Å. By this time, the F–F separation, which had previously oscillated but not exceeded 1.81 Å, extends to 2.70 Å and continues to grow toward a maximum value of 4.21 Å at the 750 fs mark (middle panel, green trace). The integrated net spin (lower panel, magenta trace) and integrated spin modulus (lower panel, black trace) oscillate in sympathy during the initial approach of the molecule to the surface, indicating incipient triplet nature, but increasingly diverge from one another from about the 400 fs mark onward. A little after the moment of closest approach, the integrated net spin falls to zero and the integrated spin modulus briefly touches 1 μ_B_ before itself abruptly falling to zero as the nascent C–F bonds form. Thereafter, both spin measures remain essentially zero, except for occasional blips, implying a complete absence of radical character.

**Figure 5:**
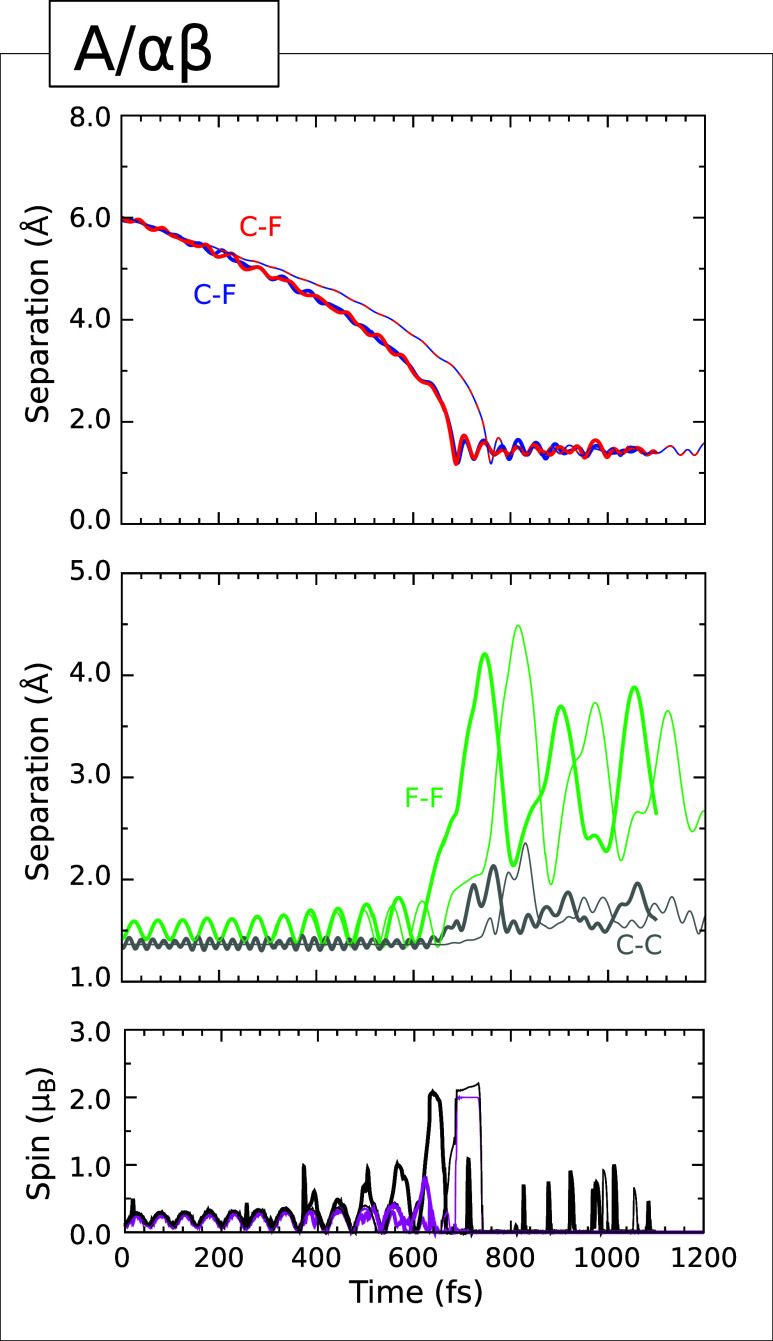
Evolution of the A/αβ (Type II) trajectory. The upper panel shows the C–F separations corresponding to the adsorption of both fluorine atoms (blue and red) at either end of a single dimer. The middle panel shows the F–F separation (green) and the C–C separation for the dimer to which the fluorine atoms become attached (gray). The lower panel shows the integrated net spin (magenta) and the integrated spin modulus (black). Across all panels, simulations performed with an initial surface temperature of 0 K (300 K) are indicated with thin (thick) traces.

After their formation, the two C–F bonds both oscillate about a common mean length of around 1.42 Å. The F–F separation, meanwhile, fluctuates in the range 2.14–3.88 Å, consistent with the location of the two adatoms at either end of a single surface dimer. After the initial disruption of the molecule’s arrival, the C–C separation associated with that dimer oscillates over the range 1.48–1.96 Å at an approximate frequency of 920 cm^–1^. This is substantially red-shifted compared with the 1489 cm^–1^ (184.6 meV) reported experimentally for the dimer stretch mode of the clean surface.^49^ Indeed, it falls squarely within the 800–1200 cm^–1^ range typically cited for C–C stretch modes in alkanes, rather than the 1500–1700 cm^–1^ range more commonly found in alkenes and other unsaturated compounds, once again supporting the interpretation that the dimer bond switches from double- to single-bond character upon adsorption of fluorine. Needless to say, the final state of the surface features no dangling bonds, since each carbon atom of the dimer is saturated with a fluorine atom. As for the simulation that started with the surface at 0 K, examination of Figure 5 reveals that the same key stages are replicated with a delay of around 65–75 fs relative to the 300 K simulation. Once again, the warm surface appears to be slightly more reactive than the cold.

### Type IIIa Trajectory

Turning now to the E/αβγ trajectory, whose evolution is displayed in Figure 6, we see an instance where each fluorine atom will end up bonded to a different surface dimer. Specifically, the two involved dimers in this case are neighbors within a single dimer row, and the fluorine atoms attach at the same end of each dimer. That is to say, they are proximal to one another, bonding as closely as possible for two adatoms that do not inhabit the same surface dimer. The C–F distances corresponding to the nascent bonds (upper panel, blue and red traces) show progressive acceleration toward the surface, similar to that described in the cases dealt with above, while the F–F separation again oscillates in response to electron transfer from the substrate (middle panel, green trace). As before, the integrated net spin (lower panel, magenta trace) and the integrated spin modulus (lower panel, black trace) oscillate in sympathy to begin with, but diverge as the molecule nears the surface, implying a transition from an incipient triplet configuration toward a more singlet-like situation.

**Figure 6:**
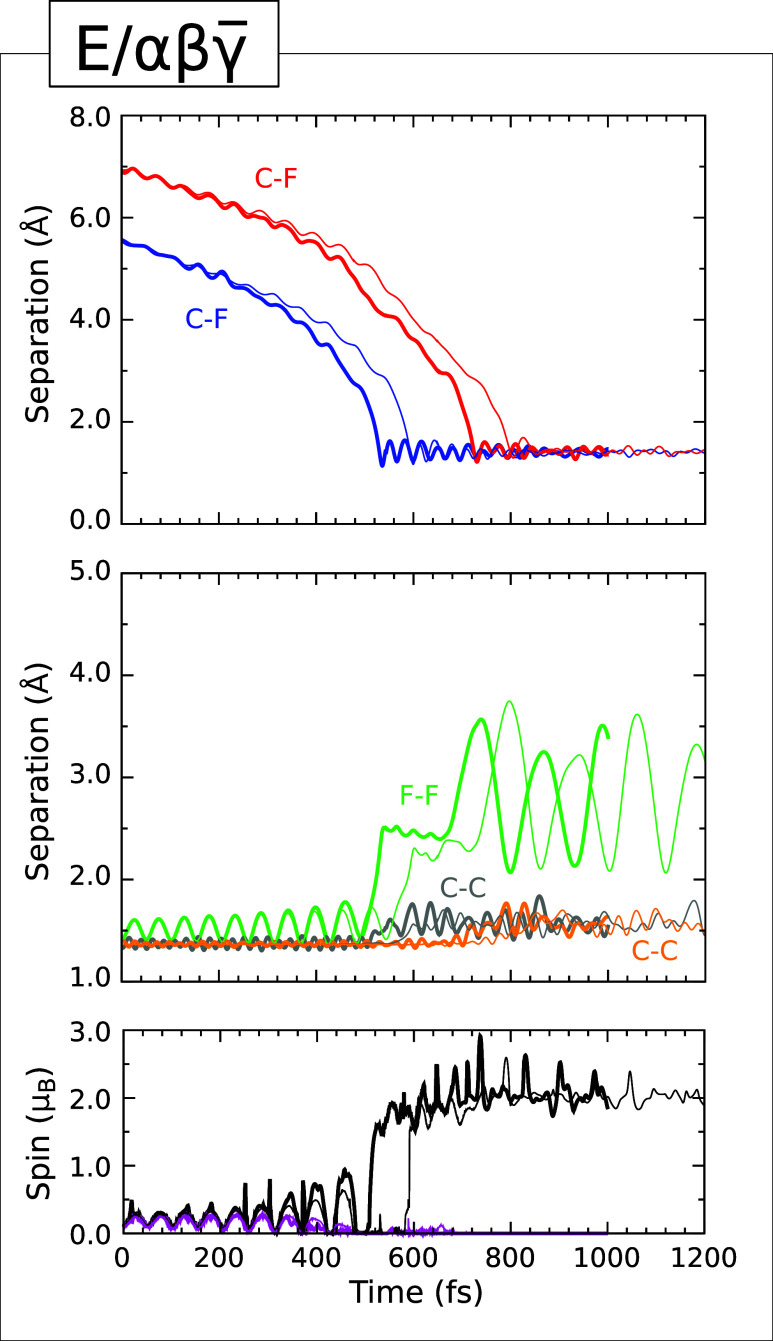
Evolution of the E/αβγ (Type IIIa) trajectory. The upper panel shows the C–F separations corresponding to the adsorption of fluorine atoms (blue and red) at the proximal ends of two adjacent dimers from the same dimer row. The middle panel shows the F–F separation (green) and the C–C separations for the two dimers to which the fluorine atoms become attached (gray and orange). The lower panel shows the integrated net spin (magenta) and the integrated spin modulus (black). Across all panels, simulations performed with an initial surface temperature of 0 K (300 K) are indicated with thin (thick) traces.

In the 300 K simulation, the crucial moment comes at the 565 fs mark, when the C–F distance involving the lower-lying fluorine atom reaches its minimum value of 1.21 Å before settling into oscillations about a mean bond length of around 1.39 Å at an approximate frequency of 1050 cm^–1^. This is not unreasonable vibrational behavior for a monofluoride (cf. the 1049 cm^–1^, 1055 cm^–1^ and 1262 cm^–1^ stretch modes reported in methyl fluoride,^50^ fluoroacetylene,^50^ and tert-butyl fluoride,^51^ for example). At the same moment, the F–F separation, which had not previously exceeded 1.78 Å, achieves a temporally local maximum value of 2.51 Å. The two spin measures, meanwhile, attain values consistent with radical character attached both to the now-isolated higher-lying fluorine atom and to the adatom-free end of the involved surface dimer (reminiscent of the end-point of the Type I trajectory described above). The C–C separation of this dimer henceforth oscillates at an approximate frequency of 885 cm^–1^ in the range 1.41–1.83 Å, consistent with conversion to a single bond (middle panel, gray trace).

Over the following 165 fs, the loose fluorine atom continues to accelerate toward the surface, reaching its point of closest approach at the 730 fs mark with a C–F distance of 1.22 Å. About 10 fs later, the F–F separation reaches a maximum value of 3.56 Å before settling back to oscillate in the 2.07–3.50 Å range. The original C–F bond is not much perturbed by the formation of the second, and both behave in similar manner to one another throughout the remainder of the simulation. The C–C separation associated with the second dimer increases, oscillating at an approximate frequency of 890 cm^–1^ within the range 1.44–1.77 Å (middle panel, orange trace). Examination of the system’s net spin density at the 800 fs mark, as displayed in Figure 7, reveals localization on both of the adatom-free carbon atoms belonging to the affected dimers, and Mulliken analysis indicates spin moments of 0.80 μ_B_ on one and −0.81 μ_B_ on the other. These results are entirely consistent with the existence of semioccupied sp^3^ dangling bonds exhibiting singlet diradical character. Indeed, we have explicitly tested a triplet diradical solution for this geometry and find it to be less stable than the singlet diradical configuration by 0.05 eV.

**Figure 7:**
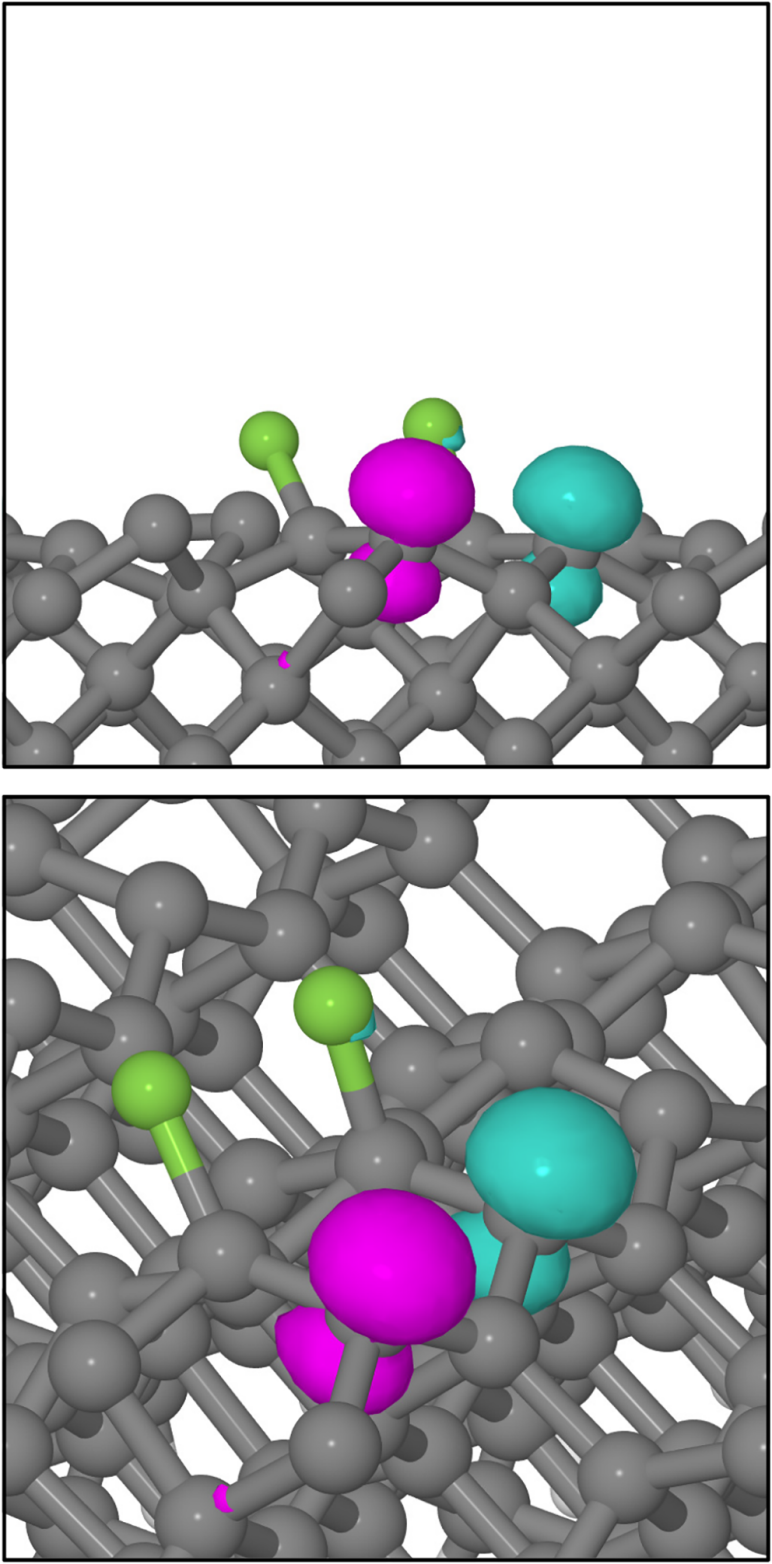
Net spin density at the end of the E/αβγ trajectory (isosurfaces plotted at 0.1 μ_B_·Å^–3^ with turquoise and magenta indicating opposing signs). The upper panel shows an orthographic projection along the [110] crystallographic direction, while the lower panel shows a view from an elevated position along a similar azimuth. Gray and green spheres represent carbon and fluorine atoms respectively. Note the sp^3^-like distributions at the two dangling bonds, coupled in singlet diradical fashion.

In line with the previously described trajectories, the simulation performed with an initial surface temperature of 0 K exhibits all of the same behaviors as are evident at 300 K, differing only in that the key events are delayed by 60–80 fs. The pattern of slightly enhanced reactivity at the warm surface compared with the cold is thus maintained.

### Type IIIb Trajectory

The evolution of key parameters in the B/αβγ trajectory, displayed in Figure 8, is rather similar to that described immediately above. Once again, two C–F separations (upper panel, blue and red traces) indicate progressive acceleration of the molecule toward the surface, and in the 300 K case the shortest of these reaches a minimum value of 1.25 Å at the 550 fs mark. By this point, the F–F separation (middle panel, green trace) has expanded to 2.46 Å and the two spin measures are well on their way toward values reminiscent of the end-point of a Type I trajectory. That is, radical character will be associated with the now-isolated higher-lying fluorine atom and with the adatom-free end of the affected surface dimer. The C–C bond length of this dimer (middle panel, orange trace) subsequently fluctuates in the range 1.45–1.75 Å, suggestive of a single bond, while the C–F bond oscillates at an approximate frequency of 1025 cm^–1^ about a mean bond length of around 1.40 Å.

**Figure 8:**
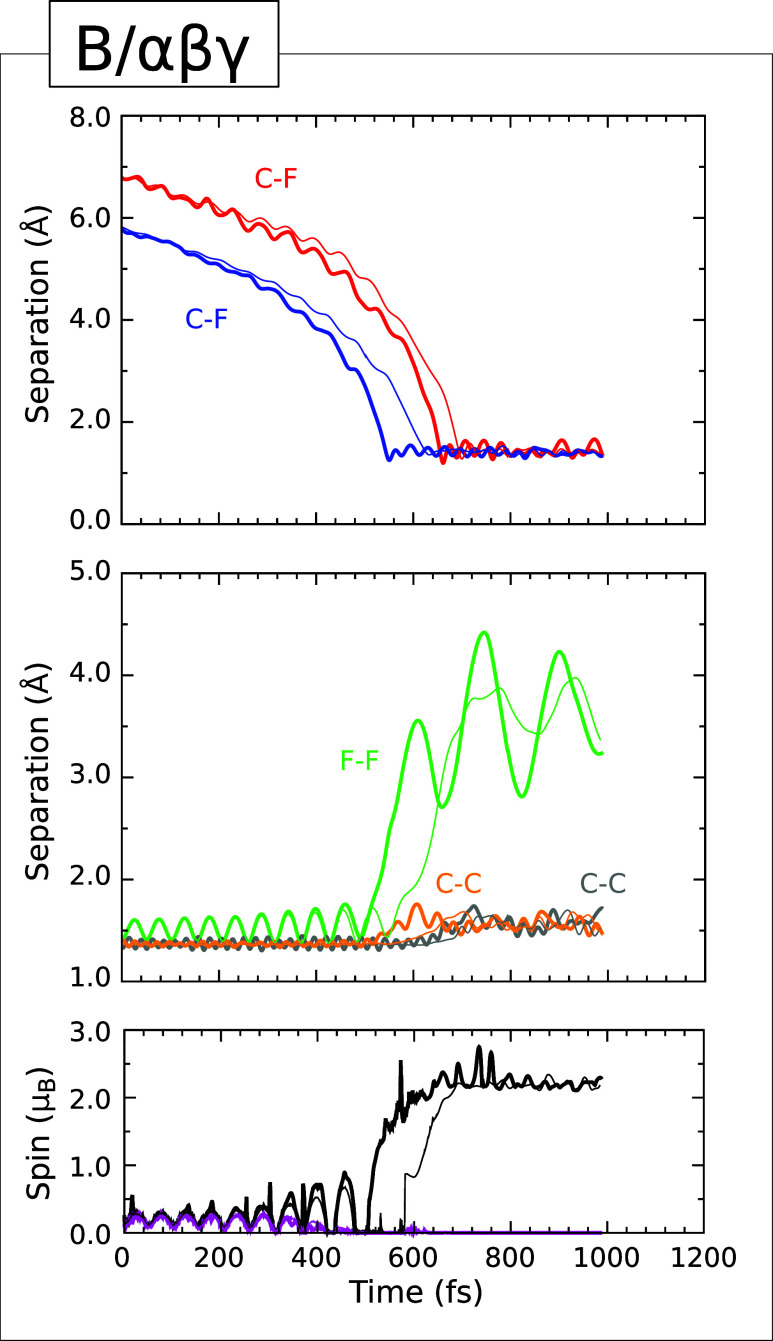
Evolution of the B/αβγ (Type IIIb) trajectory. The upper panel shows the C–F separations corresponding to the adsorption of fluorine atoms (blue and red) at the distal ends of two adjacent dimers from the same dimer row. The middle panel shows the F–F separation (green) and the C–C separations for the two dimers to which the fluorine atoms become attached (gray and orange). The lower panel shows the integrated net spin (magenta) and the integrated spin modulus (black). Across all panels, simulations performed with an initial surface temperature of 0 K (300 K) are indicated with thin (thick) traces.

A little over 110 fs after arrival of the first fluorine atom, the second C–F separation reaches its minimum value of 1.20 Å just after the 660 fs mark. The involved carbon atom belongs to a dimer adjacent to the first within a single dimer row, but lies distal to the carbon atom that hosts the first fluorine atom. Accordingly, the F–F separation fluctuates in the range 2.81–4.42 Å for the remaining duration of the simulation (cf. 2.07–3.50 Å in the Type IIIa trajectory described above) while the C–C separation associated with the second dimer oscillates in the range 1.44–1.74 Å, indicating conversion to a single bond. During this phase, the two spin measures are consistent with localization of sp^3^ dangling bonds on the two adatom-free carbon atoms of the involved dimers, implying a surface showing singlet diradical character. Explicitly testing a triplet solution at the 800 fs mark, we find it to be 0.01 eV less stable than the singlet solution at the same moment. The second C–F bond, meanwhile, oscillates in manner similar to the first, albeit with a slightly greater amplitude. At this point in our discussion, it is probably unsurprising to note that the simulation performed with an initial surface temperature of 0 K behaves in rather similar manner to the one performed with an initial surface temperature of 300 K. The delay of key events in the cold case, relative to the warm case, is rather less consistent here, however, ranging between 40 and 120 fs. Nevertheless, the enhanced reactivity of the warm surface is once again clearly in evidence.

### Type IVa Trajectory

For our next example, we turn to the C/γ trajectory, whose evolution is displayed in Figure 9. Here, the pattern familiar from the previous two trajectories seems to be followed closely until the lower-lying fluorine atom makes its closest approach to the surface, with a C–F separation of 1.25 Å at the 600 fs mark (upper panel, blue trace). Indeed, the only evident difference is that the integrated net spin (lower panel, magenta trace) and integrated spin modulus (lower panel, black trace) both approach values of 2 μ_B_ around this time. This indicates that radical character associated with the surface and the now-isolated fluorine atom must be coupled in a triplet-like manner, but it is unlikely that this has very much consequence for the subsequent behavior. Certainly the integrated net spin falls to zero at around the 685 fs mark, well before the C–F separation involving the second fluorine atom and its eventual bonding partner (upper panel, red trace) reaches its minimum value of 1.22 Å at the 725 fs mark. After this point, the F–F separation (middle panel, green trace) fluctuates in the range 2.03–3.51 Å, consistent with the fact that two adatoms bond in proximal fashion to a pair of surface dimers that are neighbors in adjacent rows. The C–C bond lengths of the two affected dimers (middle panel, gray and orange traces) both oscillate in a rather irregular manner within the range 1.43–1.69 Å during this latter portion of the simulation, suggesting single-bond character. The two spin measures are once again consistent with the presence of sp^3^ dangling bonds associated with the adatom-free carbon atoms of the involved dimers, coupled in a singlet diradical configuration. We must provide the caveat, however, that interactions with periodic images may start to become important in the latter stages of the Type IV cases, so we do not attempt to quantify the singlet–triplet splitting.

**Figure 9:**
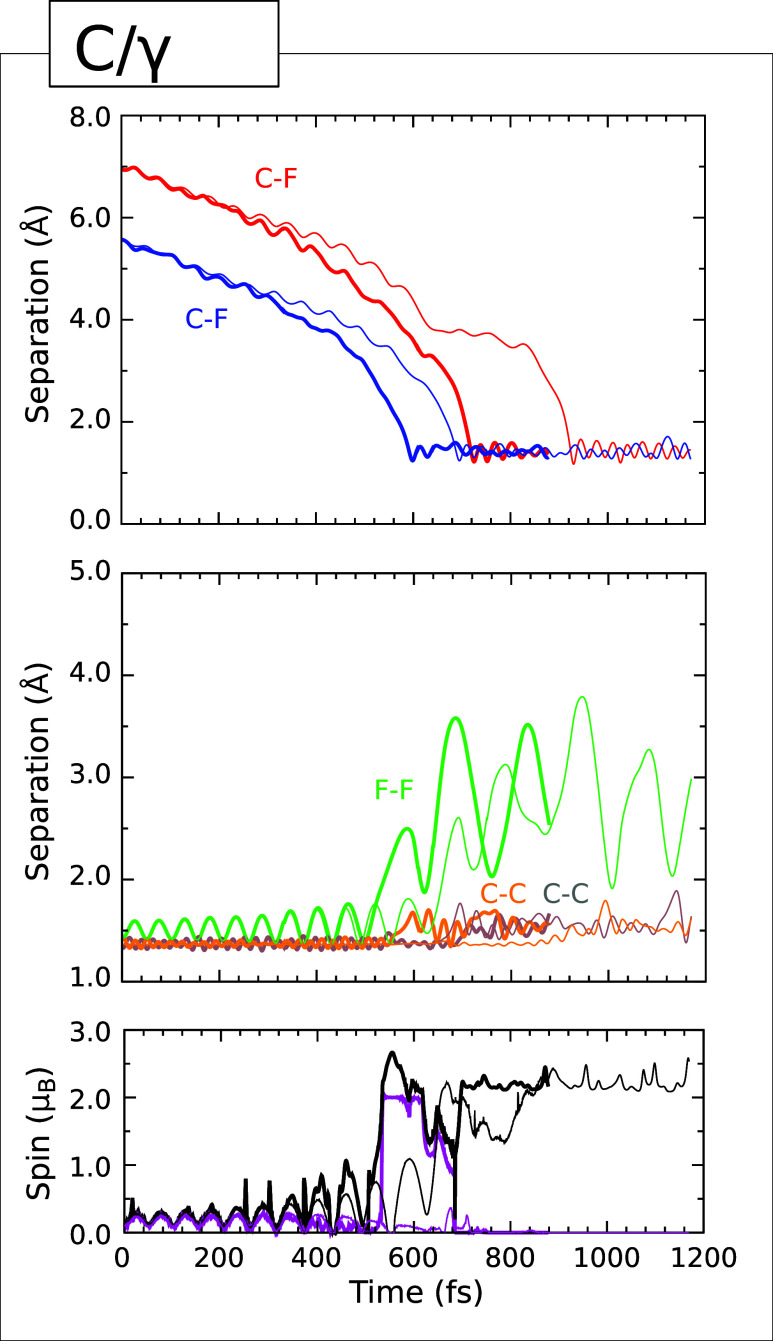
Evolution of the C/γ (Type IVa) trajectory. The upper panel shows the C–F separations corresponding to the adsorption of fluorine atoms (blue and red) at the proximal ends of two adjacent dimers from neighboring dimer rows. The middle panel shows the F–F separation (green) and the C–C separations for the two dimers to which the fluorine atoms become attached (gray and orange). The lower panel shows the integrated net spin (magenta) and the integrated spin modulus (black). Across all panels, simulations performed with an initial surface temperature of 0 K (300 K) are indicated with thin (thick) traces.

While it is undeniably true that the 0 K simulation once again represents essentially a delayed version of the 300 K simulation, the differences here are rather greater than for any of the trajectories discussed above. For instance, the moment of closest approach for the first C–F separation is delayed by just over 95 fs in the cold case relative to the warm, but that of the second C–F separation is delayed by around 205 fs. Whether this may be explained by the lack of a triplet-like interlude in the 0 K simulation is a matter for speculation.

### Type V Trajectory

Finally, we arrive at the D/αβγ trajectory, depicted graphically in Figure 10. In this case, the two fluorine atoms end up bonding with carbon atoms from two dimers that are neither neighbors within a single row nor neighbors between adjacent rows. The separation corresponding to formation of the first C–F bond (upper panel, blue trace) achieves its minimum value of 1.29 Å just after the 685 fs mark, while that corresponding to formation of the second C–F bond (upper panel, red trace) achieves its minimum value of 1.13 Å at the 750 fs mark. Both bonds subsequently oscillate at an approximate frequency of 1015 cm^–1^ about a common mean length of around 1.40 Å. The F–F separation fluctuates in the range 2.86–4.24 Å, which is comparable with the range of 2.81–4.42 Å noted above in our Type IIIb example. After adsorption, the two C–C separations (middle panel, gray and orange traces) both oscillate within the range 1.41–1.76 Å at frequencies close to 1030 cm^–1^, consistent with their conversion to single-bond character. Yet again, the two spin measures (lower panel, magenta and black traces) are consistent with the singlet diradical coupling of sp^3^ dangling bonds on the adatom-free carbon atoms of the involved dimers, but we cannot reliably determine the singlet–triplet splitting due to possible interactions between periodic images in the latter stages of Type V adsorption.

**Figure 10:**
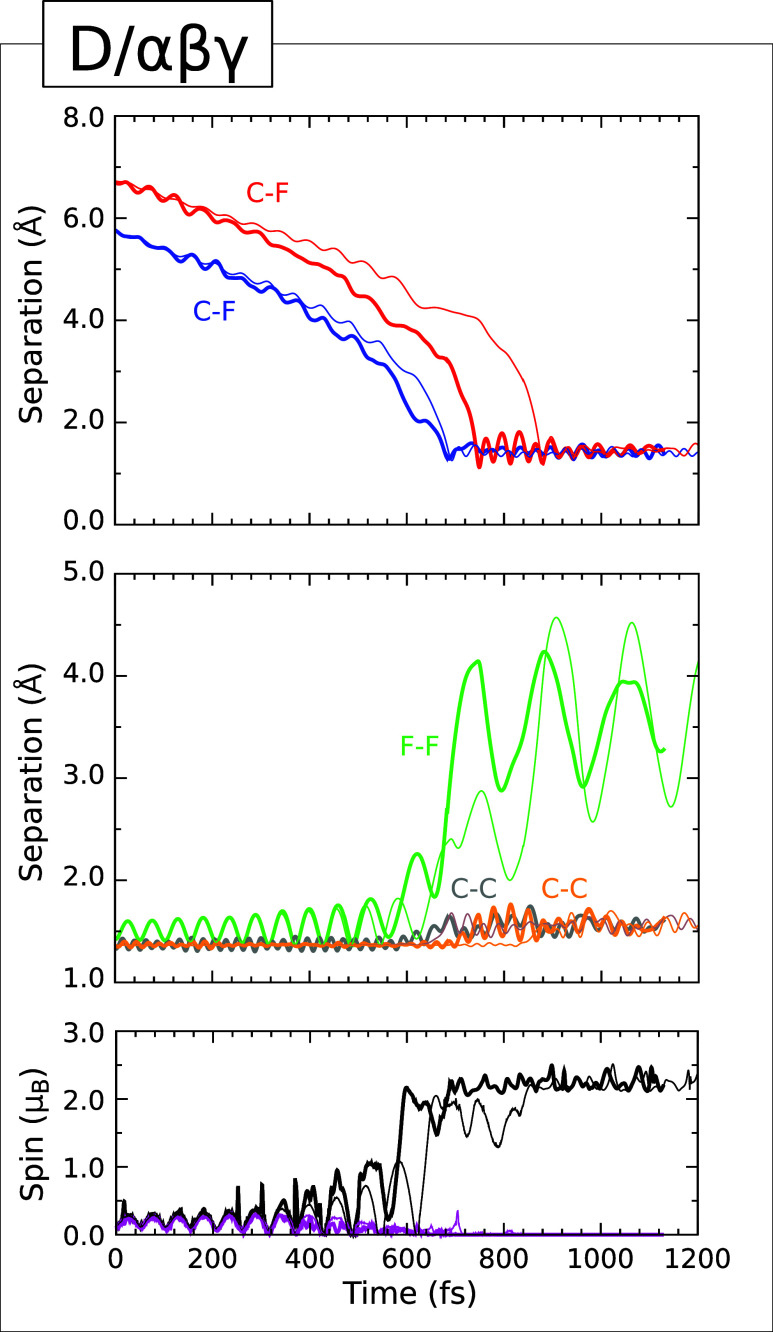
Evolution of the D/αβγ (Type V) trajectory. The upper panel shows the C–F separations corresponding to the adsorption of fluorine atoms (blue and red) at ends of two nonadjacent dimers. The middle panel shows the F–F separation (green) and the C–C separations for the two dimers to which the fluorine atoms become attached (gray and orange). The lower panel shows the integrated net spin (magenta) and the integrated spin modulus (black). Across all panels, simulations performed with an initial surface temperature of 0 K (300 K) are indicated with thin (thick) traces.

The comparison between the cold- and warm-surface simulations is a little atypical in this instance, with almost no difference in the arrival time of the first fluorine atom. The corresponding rise in the integrated spin modulus is nevertheless delayed by around 60 fs in the 0 K simulation compared with the 300 K simulation, while the arrival time of the second fluorine atom is delayed by around 130 fs. Despite this more complex picture, the greater reactivity of the warm surface relative to the cold is apparent once more.

## Conclusions

The present calculations provide insight into the adsorption of molecular fluorine on diamond at both low and moderate surface temperatures. A range of outcomes may be observed, in most of which both fluorine atoms adsorb (complete adsorption). In a substantial minority of cases, however, only a single fluorine atom adsorbs, the other being ejected whence it came (partial adsorption). The latter events confer radical character on the surface, as well as implying the same for the desorbing atom itself. An interesting comparison may be made with previous calculations of similar type for the adsorption of molecular fluorine on Si{001}.^16^ Although the ejection of radical fluorine atoms from that surface has been inferred from experimental data,^52^ predicted by molecular dynamics calculations employing empirical potentials,^53–56^ and subject to kinetic modeling,^57–59^ first-principles molecular dynamics indicated that radical fluorine would most likely be only transiently isolated prior to rapid adsorption onto the surface.^16^ Here the situation is quite different and the desorption of radical fluorine appears to be a quite plausible prospect. Nevertheless, our results indicate an effective sticking probability (half the number of adsorbing fluorine atoms per impinging fluorine molecule) of at least 0.90 in both temperature regimes.

Regarding the role of surface temperature, an increase from 0 to 300 K serves mainly to shift individual trajectories between common reaction channels rather than establishing any qualitatively new behavior. The most notable effect is to substantially enhance the probability that both fluorine atoms will bond to either end of a single carbon dimer, resulting in a closed-shell surface. Nevertheless, bonding to separate dimers remains the dominant mechanism, albeit expressed in a multitude of variations. Common to all such cases, however, is the generation of semioccupied dangling bonds coupled in a singlet diradical configuration. This unusual situation shows every sign of being stable within our calculations, at least when the fluorine atoms are closely coadsorbed, although we note that dangling bonds will doubtless serve as active sites for further adsorption. Clearly the lifetime of singlet diradical surface states will be dictated by chemical kinetics as much as by electronic considerations, and we hope this may ultimately be susceptible to experimental confirmation.

## Data Availability

The trajectories analyzed in this study are openly available in the University of Cambridge Data Repository at DOI: 10.17863/CAM.119059.
